# Bardet‐Biedl syndrome: Weight patterns and genetics in a rare obesity syndrome

**DOI:** 10.1111/ijpo.12703

**Published:** 2020-07-22

**Authors:** Jeremy Pomeroy, Anthony D. Krentz, Jesse G. Richardson, Richard L. Berg, Jeffrey J. VanWormer, Robert M. Haws

**Affiliations:** ^1^ Clinical Research Center Marshfield Clinic Research Institute Marshfield Wisconsin USA; ^2^ Prevention Genetics Marshfield Wisconsin USA; ^3^ Department of Pediatrics Marshfield Clinic Health System Marshfield Wisconsin USA

**Keywords:** Bardet‐Biedl syndrome, genetics, loss of function variants, missense variants, obesity, overweight

## Abstract

**Background:**

Bardet‐Biedl syndrome (BBS) is a rare genetic disorder that severely inhibits primary cilia function. BBS is typified by obesity in adulthood, but pediatric weight patterns, and thus optimal periods of intervention, are poorly understood.

**Objectives:**

To examine body mass differences by age, gender, and genotype in children and adolescents with BBS.

**Methods:**

We utilized the largest international registry of BBS phenotypes. Anthropometric and genetic data were obtained from medical records or participant/family interviews. Participants were stratified by age and sex categories. Genotype and obesity phenotype were investigated in a subset of participants with available data.

**Results:**

Height and weight measurements were available for 552 unique individuals with BBS. The majority of birth weights were in the normal range, but rates of overweight or obesity rapidly increased in early childhood, exceeding 90% after age 5. Weight z‐scores in groups >2 years were above 2.0, while height z‐scores approached 1.0, but were close to 0.0 in adolescents. Relative to those with the BBS10 genotype, the BBS1 cohort had a lower BMI z‐score in the 2‐5 and 6‐11 age groups, with similar BMI z‐scores thereafter. Children with biallelic loss of function (LOF) genetic variants had significantly higher BMI z‐scores compared to missense variants.

**Conclusion:**

Despite normal birth weight, most individuals with BBS experience rapid weight gain in early childhood, with high rates of overweight/obesity sustained through adolescence. Children with LOF variants are disproportionally affected. Our findings support the need for earlier recognition and initiation of weight management therapies in BBS.

**Abbreviations**
BBS: Bardet‐Biedl syndrome
BMI: body mass index
LOF: loss of function
PHI: Personal Health Information
z‐BMI: z‐score body mass index
z‐WFL: z‐score weight for length

## 1 INTRODUCTION

Childhood obesity is a dominant feature of Bardet‐Biedl syndrome (BBS; OMIM 209900), a rare, pleiotropic and multigenic ciliopathy.^1^ The centrality of obesity in the syndrome was recognized at least one century ago when the French physician Georges Louis Bardet submitted his 1920 thesis on hypothalamic obesity describing two French girls with polydactyly, obesity and retinitis pigmentosa.^2^ Hungarian pathologist‐endocrinologist Artur Biedl further defined the BBS phenotype 2 years later in his description of siblings with obesity, cognitive impairment, polydactyly, and genital anomalies.^3^ Although the mechanisms for obesity in BBS remains incompletely understood, disruption of the hypothalamic leptin‐melanocortin signaling pathway is evident, and BBS may provide insights into obesity in other monogenic and syndromic obesity disorders.^4^, ^5^

Rates of overweight and obesity in mixed populations of children and adults with BBS exceed 70%.^6^, ^7^, ^8^, ^9^ The majority of individuals with BBS have some level of excess body weight by adulthood; however, weight gain patterns in childhood and adolescence remain unclear. Systematic examination of growth patterns in early life have not been rigorously examined, and gender‐specific growth patterns are unexplored. At least 25 causative genes have been identified in BBS.^10^, ^11^, ^12^ Previous reports document that truncating variants in BBS genes predict greater risk for severe chronic kidney disease and increased cardiovascular disease markers compared to missense variants.^13^, ^14^ Similar obesity genotype‐phenotype correlations have not been explored in BBS. Rare disease registries employed to examine weight patterns and genotype‐phenotype correlations could provide further insight into rare obesity syndromes.^15^

In this study, we used a natural history registry to explore the prevalence of overweight and obesity in the largest known international pediatric BBS cohort. We examined BBS growth patterns in infancy, childhood, and adolescence. A second objective was to explore genotype and phenotype correlations with specific attention to the two most common BBS genotypes in North America, namely *BBS1* and *BBS10*. Furthermore, we explored BBS variants based on their role in primary cilia function—the cellular organelle disrupted in BBS—and the role of loss of function variants in disease expression.

## 2 METHODS

### 2.1 Source population

The Clinical Registry Investigating Bardet‐Biedl Syndrome (CRIBBS) is an open enrolling international database, established in June 2014, designed to record longitudinal health outcomes in individuals with BBS (ClinicalTrials.gov: NCT02329210). As of January 1, 2020, there were 552 individuals residing in 39 countries enrolled in CRIBBS. Individuals with clinical features meeting established diagnostic criteria for BBS were included in the present analysis.^6^ BBS genotype and height/length and weight measurements during childhood (<20 years old) were available in 343 individuals. Prior to inclusion in CRIBBS, informed consent was obtained from enrollees or their legal guardians. All registry procedures were approved by the Marshfield Clinic Health System Institutional Review Board.

### 2.2 Measures

#### 2.2.1 Anthropometrics

A senior research coordinator collected self‐reported height/length and weight measurements during annual health interviews with study participants. In addition, research team members extracted height/length and weight measurements from medical records obtained in compliance with the Health Insurance Portability and Accountability Act. If both medical record and self‐reported data were available for a patient on the same date, the medical record data were used. Birth weight was evaluated using the World Health Organization (WHO) Child Growth Standards^16^ for infants born at term. Birth weights between the 10th and 90th percentile were considered normal. Births prior to 37 weeks gestation were classified as preterm births. Preterm births were further subdivided into extremely preterm (less than 28 weeks), very preterm (28 to 32 weeks), and moderate to late preterm (32 to 37 weeks) using WHO criteria. For preterm births, birth weight for gestational age was evaluated using the reference values presented by Oken et al.^17^

#### 2.2.2 Genetic variants

The BBS diagnosis was supported by genetic testing in 385 CRIBBS participants. Seventeen different BBS genes were represented and listed according to descending frequency—*BBS1*, *BBS10*, *BBS2*, *MKKS/BBS6*, *BBS7*, *BBS9*, *BBS12*, *BBS4*, *ARL6/BBS3*, *BBS5*, *BBS8*, *BBIP1/BBS18*, *CEP164*, *CEP290*, *IFT140*, *IFT172*, *TTC21B*. In‐frame deletions and in‐frame duplication variants were combined with missense variants in this study. Nonsense, frameshift, copy number variants (deletions of one or more exons), and splicing variants were considered loss‐of‐function (LOF) variants. There were 224 participants with pathogenic variants in *BBS1*, *BBS2*, *BBS4*, *BBS5, BBS7*, *TTC8*, *BBS9* and *BBIP1/BBS18* genes, which are known to assemble into the BBSome.^18^ There were 116 participants with pathogenic variants in *MKKS/BBS6*, *BBS10* and *BBS12* genes, which make up the chaperonin complex.^19^

### 2.3 Statistical Analyses

Data were analyzed using SAS version 9.4 (SAS Institute Inc., Cary, North Carolina). Participants were stratified by sex and grouped by age, with groups consisting of <2 years (not including birth weight), age 2‐5 years, 6‐11 years and 12‐19 years. When a participant had more than one height/length and weight measurement within an age group, the median z‐score was calculated and used in the analysis. Frequencies of overweight and obesity were calculated using both the WHO Child Growth Standards^16^ and the International Obesity Task Force (IOTF) BMI cut‐offs.^20^ WHO standards define overweight as >2 SDs above the WHO Growth Standards median of weight‐for‐length for children under 5 years‐of‐age and > 1 SD above the WHO Growth Standards median BMI‐for‐age for children ages 5 to 19. Obesity is defined as >3 SDs above the WHO Growth Standards median for weight‐for‐height for children under 5 years‐of‐age and >2 SDs above the WHO Growth Standards median BMI‐for‐age for children ages 5 to 19. The IOTF uses BMI cut‐offs for age and sex that correspond to adult overweight (BMI > 25 kg/m^2^) or obesity (BMI > 30 kg/m^2^). BMI z‐scores (zBMI) were calculated using the WHO LMS method.^21^ Comparisons of zBMI between boys and girls, as well as by genotypes, variant types, and protein groups, were evaluated with Kruskal‐Wallis nonparametric tests stratified by age group. Two‐tailed p values were considered significant at <0.05., without adjustment for multiple comparisons.

## 3 RESULTS

This analysis included 453 length and weight measurements in 119 participants less than 2 years of age (not including birth weight), and height and weight measurements in 509 participants 2 years of age and older. We included 3674 height or length and weight measurements overall.

### 3.1 Birthweight

Weight at birth was available for 510 participants, with 449 (88%) births between 37 and 42 weeks gestation. Of the 449 term births, the median (25th, 75th) birth weight was 3538 g (3221 g, 3856 g). The majority of birth weights were normal with 376 (84%) under 4000 g. Of the 73 participants with a birth weight greater than 4000 g, 58 (79%) had a birth weight of 4000‐4499 g, 14 (19%) had a birth weight of 4500‐4999 g, and 1 participant (1.3%) had a birth weight of 5000 g or greater.

Of the 61 preterm births, 54 were moderately preterm (32 to 37 weeks), 6 were very preterm (28 to 32 weeks) and 1 was extremely preterm (less than 28 weeks). The majority of preterm births were appropriate for gestational age (69.5%), including 71.7% of moderately preterm and 50% of very preterm births. Large for gestational age occurred in 28.3% of the moderately preterm and 50% of the very preterm births. Small for gestational age occurred in 1 (1.9%) moderately preterm birth and in the only extremely preterm birth.

### 3.2 Overweight/Obesity

The participants in each age group who had obesity, overweight, or did not have obesity or overweight were determined using the WHO Child Growth Standards (Table 1A) and IOTF Standards (Table 1B). In categories above age 2 years, obesity is common using either standard. Only the WHO standards include reference for children under the age of 2. In those under 2 years, 23% of participants had obesity and 56% had overweight or obesity. The prevalence of obesity or overweight increased in the 2‐5 age group to 79%, with 60% having obesity. Over 70% of all age groups had obesity using the IOTF standards with the prevalence of overweight or obesity ranging from 86% to 95%.

**TABLE 1:** Prevalence of obesity and overweight stratified by sex and age group

Age Group (years)	Girls			Boys			All		
	Without overweight/obesity	Overweight	Obesity	Without overweight/obesity	Overweight	Obesity	Without overweight/obesity	Overweight	Obesity
**A. WHO Child Growth Standards**									
< 2^a^	21 (42.0)	18 (36.0)	11 (22.0)	32 (46.4)	21 (30.4)	16 (23.2)	53 (44.5)	39 (32.8)	27 (22.7)
2–5	19 (20.9)	24 (26.4)	72 (52.7)	23 (21.9)	12 (11.4)	70 (66.7)	42 (21.4)	36 (18.4)	118 (60.2)
6–11	5 (5.0)	13 (12.9)	82 (82.2)	8 (6.7)	11 (9.2)	101 (84.2)	13 (5.9)	24 (10.9)	184 (83.3)
12–19	5 (5.2)	15 (15.5)	76 (79.4)	12 (10.8)	16 (14.4)	83 (74.8)	17 (8.2)	31(14.9)	160 (76.9)
**B. IOTF Child Growth Standards**									
2–5	12 (13.2)	15 (16.5)	64 (70.3)	16 (15.2)	14 (13.3)	75 (71.4)	28 (14.3)	29 (14.8)	139 (70.9)
6–11	5 (5.0)	20 (19.8)	76 (75.2)	12 (10.0)	24 (20.0)	84 (70.0)	17 (7.7)	44 (19.9)	160 (72.4)
12–19	6 (6.2)	19 (19.6)	72 (74.2)	13 (11.7)	24 (21.6)	74 (66.7)	19 (9.1)	43 (20.7)	146 (70.2)

*Note*: Data expressed as n (%).

^a^ The less than 2 years age group does not include birth weight. Without overweight/obesity, overweight, and obesity defined using the World Health Organization weight status categories^16^ (Table 1A) and the International Obesity Task Force^20^ (Table 1B). Weight status categories are based on zWFL for less than 2 years age group and zBMI for ages 2 and above.

### 3.3 Length or height z‐scores by age group

**FIGURE 1:**
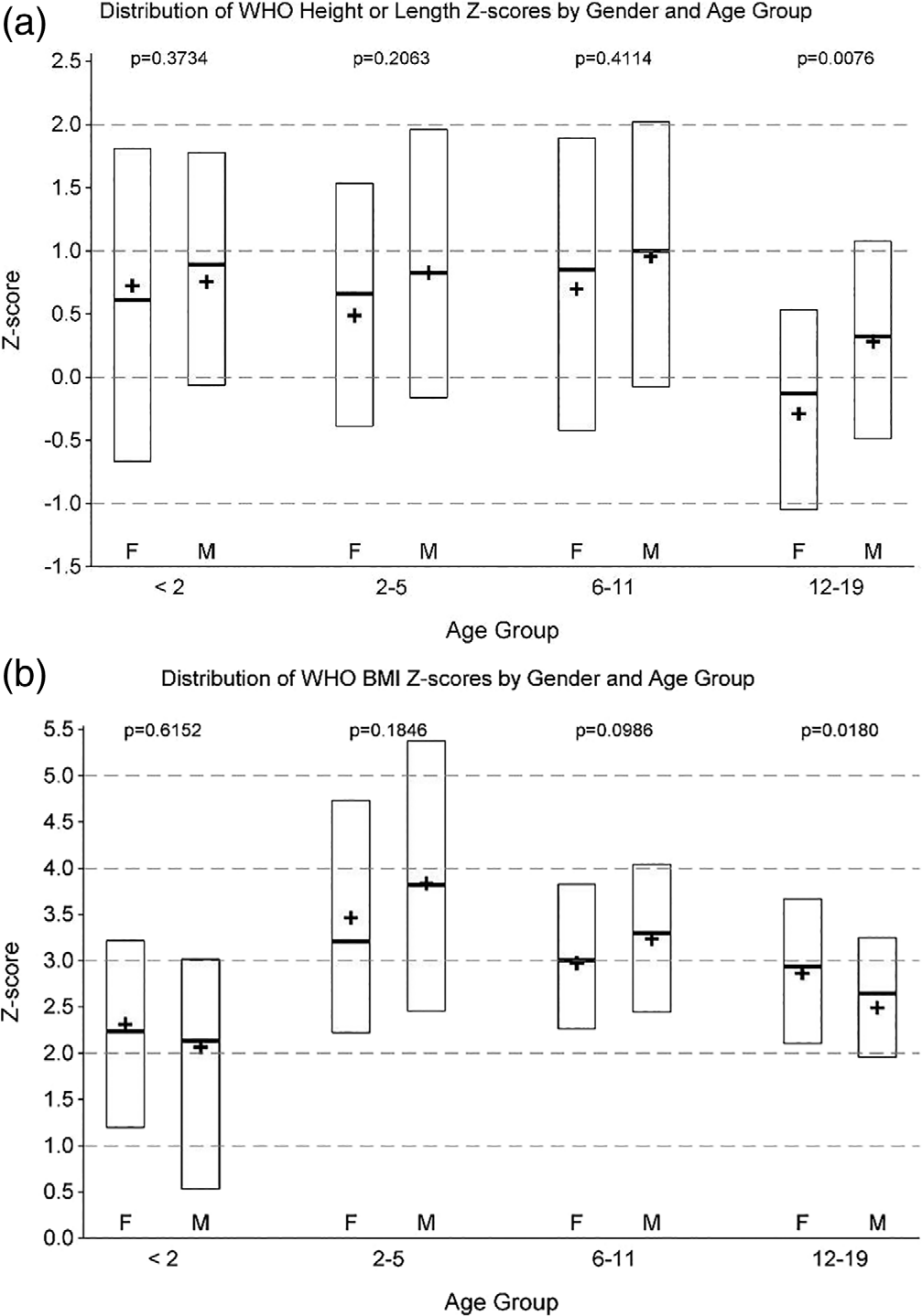
Distribution of WHO A, height or length Z‐scores and B, BMI Z‐scores by gender and age group

The length or height z‐scores by age group are shown in Figure 1A. The height z‐scores for all age groups <12 years were above average, with the median length or height z‐scores ranging from 0.5 to 1 and no significant differences between girls and boys. The height z‐scores for the 12‐19 age group were closer to 0. There was a significant difference in height z‐scores between girls and boys for the 12‐19 any age group; boys had significantly higher z‐scores than girls did (Kruskal‐Wallis *P* = .0076).

### 3.4 Body mass index z‐scores by age group

The BMI z‐scores by age group are shown in Figure 1B. The median z‐scores for all age groups were above 2. The highest median z‐scores were in the 2‐5 age group. The only between sex difference was in BMI z‐scores for the 12‐19 age group; girls had significantly higher z‐scores than boys did (Kruskal‐Wallis *P* = .0180).

### 3.5 Body mass index scores in BBS1 and BBS10 cohorts

**FIGURE 2:**
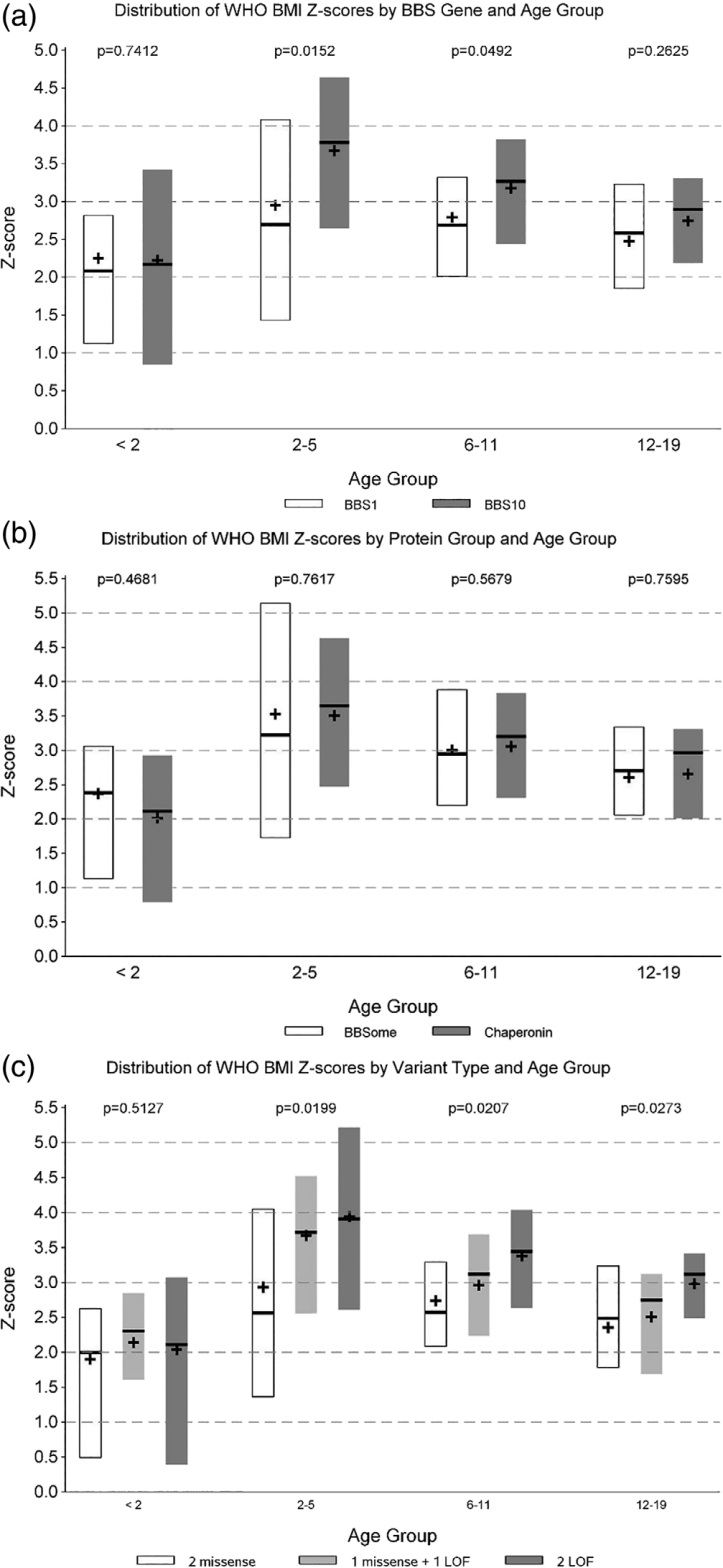
Distribution of WHO BMI Z‐scores by A, BBS gene and age group; B, protein group and age group; and C, variant type and age group

Biallelic pathogenic variants in *BBS1* (n = 144) and *BBS10* (n = 86) were the most common cause of BBS in our cohort. Comparisons of zBMI by age group, in patients with pathogenic variants in *BBS1* and *BBS10*, are shown in Figure 2A. The median BMI z‐scores were over 2 for all age and genotype groups. The only significant differences between *BBS1* and *BBS10* were in the 2‐5 years (*BBS1* 2.7 median zBMI, *BBS10* 3.8 median zBMI, Kruskal‐Wallis *P* = .0152) and the 6‐11 years (*BBS1* 2.7 median zBMI, *BBS10* 3.3 median zBMI, Kruskal‐Wallis *P* = .0492) age groups. In the 12‐19 years age group, no significant difference in zBMI (Kruskal‐Wallis *P* = .2881) was found.

### 3.6 Body mass index scores in BBSome vs BBS Chaperonin‐like Proteins

Comparisons of zBMI by age group and protein group are shown in Figure 2B. There were no significant differences for any age group by BBSome or chaperonin protein group (Kruskal‐Wallis range *P* = .4681‐.7617).

### 3.7 Body mass index scores and variant classification

Comparisons of z‐score for BMI by age group and variant type are shown in Figure 2C. BBS is usually inherited in an autosomal recessive manner. Individuals with two pathogenic variants in one BBS gene were used for this analysis. There were significant differences within each age group age 2 and older. In ages 2‐5 years (Kruskal‐Wallis *P* = .0119), individuals with two missense variants had the lowest zBMI (median 2.6). Individuals with two LOF variants (median 3.9), and individuals with one LOF and one missense variant (median 3.7) had a similar zBMI. In ages 6‐11 years (Kruskal‐Wallis *P* = .0207), two missense variants were associated with the lowest zBMI (median 2.6), with two LOF variants the highest zBMI (median 3.4), and one LOF and one missense variant falling between (median 3.1). In age 12‐19 years (Kruskal‐Wallis *P* = .0273), two missense variants were associated with the lowest zBMI (median 2.5), although individuals with one missense and one LOF variant were similar (median 2.7). Individuals with two LOF variants had the highest zBMI (median 3.1).

## 4 DISCUSSION

We examined the growth and obesity patterns in the largest international cohort of children with the rare obesity syndrome, BBS. As expected, we confirmed that obesity is a highly prevalent disease phenotype in this cohort, but several new insights were observed. Intrauterine growth appeared normal in this population, with the majority of infants delivered at term with a birth weight <4000 g. Rapid weight gain ensued during early childhood though. Of BBS children, >55% were overweight or with obesity by 2 years‐of‐age, and accelerated weight gain was particularly evident in pre‐school ages. This very early onset of obesity observed in BBS children <6 years old is atypical of childhood obesity and should raise consideration of diagnostic screening for BBS and other rare obesity syndromes.

Obesity and overweight was ubiquitous across the pediatric life span and was present in more than 90% of individuals with BBS over 6 years‐of‐age. Like common childhood obesity, children with BBS in the 2‐5 age group and the 6‐11 age group tended to be somewhat taller than average, with median height z‐scores ranging from 0.7 to 1.0, but height is largely normalized by adolescence.^22^ Body mass index z‐scores, however, persisted at a level >2 SDs above the median of the WHO reference dataset. Interestingly, BMI z‐scores improved somewhat in teenage males with BBS, but remained unchanged in females, which may warrant further research to obesity severity differences by gender in adulthood.

The present study offers valuable insight for clinicians providing care for children suspected to have genetic or syndromic obesity. We have documented that the weight patterns in infants and children with BBS are characterized by the early onset of significantly elevated BMI z‐scores. Prader‐Willi syndrome (OMIM 176270), the most frequent obesity syndrome, is characterized by failure to thrive, lack of appetite and hypotonia during infancy with excessive weight gain not being identified until after 2‐3 years of age.^23^ Likewise, failure to thrive during infancy is observed in other forms of syndromic obesity including Smith‐Magenis syndrome (OMIM 182290), Cohen syndrome (OMIM 216550), Borjeson‐Forssman‐Lehmann syndrome (OMIM 301900), Temple‐Baraiser syndrome (OMIM 616816) and a variety of disorders with chromosome deletions such as 1p36, 2q37, 6q16, 9q34 and 7p11.2.^24^, ^25^, ^26^, ^27^, ^28^, ^29^ Individuals with Alström syndrome (OMIM 203800), a ciliopathy with significant overlap with BBS, are often shorter than age matched peers,^30^ whereas the average length and height z‐score in this report and previous reports is within a normal to above average range in children with BBS. These patterns, while helpful, underscore the need to consider comprehensive evaluation, including genetic investigation, in children exhibiting early onset obesity.^31^

Our study also provides insight into the correlation of genotype and phenotype in a rare, genetically heterogeneous obesity syndrome. Previous investigators suggested a milder obesity phenotype in *BBS1* compared to other BBS genotypes.^8^, ^9^, ^32^ The present study confirms lower BMI z‐scores during childhood in the *BBS1* cohort compared to the *BBS10* cohort; however, the difference abates by adolescence. *MKKS/BBS6*, *BBS10* and *BBS12* are collectively designated as chaperonin‐like BBS proteins because of their important role in the initial assembly steps of the BBSome, a protein complex essential to primary cilia genesis and function. The BBS chaperonin‐like proteins have been linked with a more severe phenotype^19^; however, no significant difference in obesity was identified in the BBSome cohort compared to the BBS chaperonin‐like cohort. Missense or splicing variants are present in essentially all individuals with *BBS1* variants. The p.Met390Arg missense variant in *BBS1* gene is the most common variant identified in European and North American populations, while LOF variants predominate in *BBS2* and *BBS10* genotypes. Together with *BBS1*, these genotypes are identified in more than 50% of affected individuals. In the present study, we have examined three BBS cohorts: individuals with biallelic missense variants, one missense and one LOF variant, and biallelic LOF variants. We found that the severity of obesity during childhood correlated with the type of variants identified. Interestingly, we identified in CRIBBS three *BBS10* cases with biallelic missense variants. One individual died in early childhood; the other two individuals did not exhibit an obese phenotype in childhood (subject 1—BMI 23.6 kg/m^2^ at 18 years‐old and subject 2—BMI 25.6 kg/m^2^ at 14 years‐old). Individuals with BBS and LOF variants reportedly have a more severe renal phenotype and increased cardiovascular risk factors than individuals with missense variants.^13^, ^14^ Our results show that individuals with LOF variants have more severe obesity than individuals with missense variants.

Parents of children with BBS find obesity and hyperphagia to be two of the most distressing features of BBS. Oftentimes, parents utilize measures such as locking cabinets or refrigerators to limit the child's access to food. Parental guilt and an awareness of stigmatization by extended family or peers, as well as a perception of being devalued and judged as incompetent by healthcare providers, is frequently reported.^33^ The median age at diagnosis among participants in CRIBBS is 5.8 years. Increased healthcare provider awareness of rapid weight gain in early childhood BBS may facilitate earlier diagnosis, allay parental guilt, and improve the impact of therapeutic counseling. Therapeutic interventions, including behavioral therapies, may be highly effective during early childhood before diet and activity habits are established.^34^, ^35^ Obesity accelerates long‐term, co‐existing health concerns in BBS, including cardiovascular disease, obstructive sleep apnea, non‐alcoholic fatty liver disease, diabetes mellitus and negative effects on quality of life.^33^, ^36^, ^37^, ^38^, ^39^, ^40^ Vision loss resulting from retinal degeneration, poor balance or ataxia, and musculoskeletal disorders including hip dysplasia, talipes equinovarus, and scoliosis all hamper activity in children with BBS, thus aggravating the effects of underlying hyperphagia and obesity. At present, only four published case reports describe successful weight loss in BBS patients, including gastric bypass surgery, alternative bariatric procedures, and strict adherence to a diet low in protein and calories.^41^, ^42^, ^43^, ^44^ The paucity of reported successful treatments suggests that reported interventions have limited efficacy in the BBS population. Likewise, pharmacology therapy has been unsuccessful; however, recognition of the disruption of the hypothalamic leptin‐melanocortin pathway in BBS offers the potential for targeted therapy. Current clinical trials with a melanocortin‐4 receptor agonist may provide targeted therapy in children with BBS.^45^, ^46^, ^47^

Strengths of this study include the largest sample to date of children with a rare obesity syndrome and the inclusion of different countries representing a range of socioeconomic environments. We have examined a large variety of BBS genotypes generating important questions about molecular mechanisms mediating syndromic obesity. Our study examining childhood weight patterns in BBS has limitations. Obesity is a multi‐factorial disease, even in syndromic and monogenic disorders.^5^, ^31^, ^34^ Potential contributing factors such as socioeconomic, cultural, and racial parameters were not examined in this study. The impact of disease identification resulting in lifestyle modification was not examined in this report. Likewise, appetite altering medications and co‐morbidities—including diabetes mellitus and solid organ transplantation—were not considered. Type 1 diabetes was present in only one participant, while type 2 diabetes is uncommon prior to adulthood in the BBS population, unlike Alström syndrome.^30^ We have previously reported the accelerated obesity trends in renal transplant recipients with BBS.^48^ The present study utilized height/length and weight measurements primarily obtained at healthcare encounters, with some self‐reported values, and do not reflect the precision in single center prospective studies. Finally, our study examined only biallelic BBS variants and did not capture the potential additive effect of other ciliopathy genes or other obesity‐associated genes that may modulate disease severity.

In conclusion, obesity is a disease of dominant concern in the health of children with BBS. BBS growth patterns appear to be characterized by appropriate gestational length and weight, followed by disproportionate weight gain with rapid onset of overweight or obesity in early childhood and persisting through adolescence. Children with loss of function variants in BBS genes appear to be at the highest risk for severe obesity, consistent with other BBS phenotypes previously described. Our findings support the importance of exploring effective obesity therapies in individuals with BBS during early childhood.

## CONFLICT OF INTEREST

Robert Haws, MD is a consultant for Rhythm Pharmaceuticals and Trinity Life Sciences. He is a principal investigator for the Setmelanotide Phase 2 Treatment of Obesity in Rare Genetic Disorders. (ClinicalTrials.gov Identifier: NCT03013543); Setmelanotide (RM‐493), Melanocortin‐4‐Receptor Agonist, in Bardet‐Biedl Syndrome and Alström Syndrome Patients with Moderate to Severe Obesity (ClinicalTrials.gov Identifier NCT03746522) sponsored by Rhythm Pharmaceuticals and a principal investigator for A Research Study on How Well Semaglutide Works in Adolescents with Overweight or Obesity (ClinicalTrials.gov Identifier: NCT04102189) sponsored by Novo Nordisk Pharmaceuticals. Jeremy Pomeroy has received research support from Rhythm Pharmaceuticals as a co‐investigator for the Setmelanotide Phase 2 Treatment of Obesity in Rare Genetic Disorders. (ClinicalTrials.gov Identifier: NCT03013543). No other COI is declared.
